# Medulla oblongata and NCCs are central defenders against *Streptococcus agalactiae* infection of the tilapia brain

**DOI:** 10.3389/fimmu.2024.1442906

**Published:** 2024-07-01

**Authors:** Xitan Hou, Qi Li

**Affiliations:** ^1^ Institute of Forensic Medicine and Laboratory Medicine, Jining Medical University, Jining, China; ^2^ College of Fishery, Guangdong Ocean University, Zhanjiang, China

**Keywords:** Nile tilapia, *Streptococcus agalactiae*, medulla oblongata, NCCs, immune response

## Abstract

Various types of professional immune cells first emerge in fish and likely represent the primordial form and functions. Recent advancements revealed the direct connection between the central nervous system and the immune system in the mammalian brain. However, the specifics of brain-immune networks in the fish and the underlying mechanisms of teleost’s brain against pathogen infection have not been fully elucidated. In this study, we investigated the distribution of markers representing cerebral cells associated with protection and professional lymphocytes in the seven major components of the Nile tilapia brain through RNA-Seq assay and observed the most dominant abundance in the medulla oblongata. The subsequent challenge test revealed the non-specific cytotoxic cells (NCCs) exhibited the strongest response against streptococcal infection of the brain. The presence of NCCs in the brain was then confirmed using immunofluorescence and the cytotoxic effects usually induced by NCCs under infection were determined as well. Collectively, these findings contribute significantly to comprehending the mechanism of fish neuroimmune interaction and enhancing our understanding of its evolutionary development.

## 1 Introduction

Since the early 19th century, the brain has been considered a site with limited immune activity and is maintained by the blood-brain barrier (BBB) (1–4). Additionally, various cerebral cells contribute to brain homeostasis and protection (3, 5). For example, microglia function as resident myeloid cells (6), while astrocytes are involved in neuroinflammation and neurodegeneration (7, 8), serving as antigen-presenting cells and participating in inflammatory responses (3, 8). However, recent advancements have expanded our understanding of brain immunity (3, 9–11). This includes the discovery of lymphatic vessels in the brain, the identification of immunological niches, and the recognition of complex brain-immune networks. Additionally, multiple immunological niches have been found in different brain regions (3, 10, 12), such as the choroid plexus (CP), meninges, and cerebrospinal fluid (CSF). These areas contain various types of professional or peripheral immune cells (3, 13), including monocytes (Mo), natural killer cells (NKs), T cells, B cells, and dendritic cells (DCs).

In comparison to the understanding of the evolution of the nervous system in the fish brain, which shows many shared ancestral neural traits among all vertebrates (14), there has been a growing recognition of the critical role of fish immunity due to their position at the intersection of the innate and adaptive immune systems (15–17). The adaptive immune system in fish is notably simpler (16, 18), lacking structures such as bone marrow, lymph nodes, and germinal centers, as well as confirmed class switching of immunoglobulins and memory ability. Despite extensive research on the structure and neurobiological functions of fish brains (14, 19), as well as recent studies on the interactions between the nervous and immune systems in fish (20), the confirmed existence, distribution characteristics, and response patterns of professional immune cells in fish brains against stimulation remain unclear.

Therefore, in this study, we aimed to assess the distribution of immune-related cells in the tilapia brain and identify the cerebral and professional defenders involved in bacterial infection induced by *Streptococcus agalactiae*, which was well-recorded in the fish neuro-immune study since this bacterial infection usually leads to typical meningitis that was hallmarked by the clinical phenotype such as exophthalmia (21–24). Our findings revealed that cerebral cells associated with protection, as well as professional lymphocytes, were predominantly located in the medulla oblongata of the fish brain. Subsequent evaluation under challenge testing demonstrated that the NCC population exhibited the strongest response, as confirmed by the presence of NCCRP1-positive cells and assessments of cytotoxicity. Collectively, these results indicate that the central antibacterial immunity of fish brains occurs in the medulla oblongata and is mediated by NCCs.

## 2 Materials and methods

### 2.1 Fish and brain sample collection

Tilapia specimens weighing approximately 100 ± 10 grams were obtained from Zhanjiang City, China. These specimens were acclimated in recirculating aquaculture systems with appropriate ventilation for 60 days, maintaining a constant temperature of 28°C throughout the acclimation process. Tilapia were fed with commercial feed daily from Guangdong Yuehai Feeds Group Co., Ltd. (Product code 1718). Three fish were collected post the acclimation period (at 61^st^ day), anesthetized using tricaine methanesulfonate (MS-222; Sigma, Darmstadt, Germany), and then euthanized. The entire brain was carefully dissected, and several distinct brain components (25), including the olfactory bulb, cerebrum, optic lobe, cerebellum, hypothalamus, and medulla oblongata, were sampled for RNA extraction. Additionally, the thin medulla oblongata was divided into two segments along the anterior-posterior axis, referred to as the anterior medulla oblongata and posterior medulla oblongata (**Supplementary Figure S1**).

### 2.2 RNA extraction, RNA-Seq, and bioinformatics analysis

Total RNA was extracted from each sample using RNAiso Plus from TaKaRa (Dalian, China), and subsequently treated with RNase-free DNase I from TaKaRa (Dalian, China) to eliminate any residual DNA. The quality of the total RNA was confirmed by electrophoresis using 1.2% agarose gels and quantified with NanoDrop 2000 from Thermo Fisher Scientific (Waltham, USA). The mRNA molecules were enriched using oligo(dT) beads (Qiagen, Hilden, Germany), fragmented into short fragments, and subsequently reverse-transcribed into cDNA. The resulting cDNAs were collected, subjected to end repair, ligated with Illumina sequencing adapters, amplified through PCR, and subsequently sequenced using the Illumina NovaSeq 6000 platform (Gene Denovo Biotechnology Co., Guangzhou, China). The raw reads were filtered to obtain high-quality clean reads using fastp (version 0.18.0). Paired-end clean reads were mapped to the reference genome (http://ftp.ensembl.org/pub/release-110/fasta/oreochromis_niloticus/) using HISAT2 (version 2.4.0).

Gene expression abundances were calculated and normalized to fragments per kilobase of transcript per million mapped reads (FPKM). Differentially expressed genes (DEGs) were identified using DESeq2 (version 1.26.0) with the following criteria: |log2(foldchange)| ≥ 1, *P* value < 0.05, and false discovery rate (FDR, Q value) ≤ 0.05. Kyoto Encyclopedia of Genes and Genomes (KEGG) pathway enrichment analyses were conducted through the KEGG Automatic Annotation Server (KAAS).

In addition, based on our previous study on the characterization of tilapia head kidney leukocytes (HKLs) through RNA-Seq in 2022 (25), the geometric mean of three universal housekeeping genes*—β-actin*, *ef1a*, and *gapdh* (26–28)—were used to normalize the expression abundance of both the HKL and brain cDNA libraries.

### 2.3 Bacteria and challenge

The preserved *S. agalactiae* strain ZQ0910 (29) of serotype III was reactivated by incubation in brain-heart infusion broth at 28°C overnight. The bacterial culture was then harvested through centrifugation at 4000 × g for 5 minutes. The bacterial cells were subjected to three successive washes with phosphate-buffered saline (PBS) and were ultimately resuspended in PBS for subsequent experiments.

A total of 20 fish were intraperitoneally injected with 100 μL of *S. agalactiae* (5 × 10^7^ CFU/mL). Afterward, three parallel individuals were collected and sacrificed at 0, 12, and 24 hours postinfection (hpi), and brain samples were obtained using a previously described protocol for RNA extraction and cDNA synthesis.

### 2.4 cDNA synthesis and quantitative real-time PCR

cDNAs were synthesized using the PrimeScript™ RT reagent kit with gDNA Eraser from TaKaRa (Dalian, China). Quantitative real-time PCR (qRT−PCR) was performed with TB Green^®^ Premix Ex Taq™ II (Tli RNaseH Plus) from TaKaRa (Dalian, China) and the QuantStudio 6 Flex Real-Time PCR System from Thermo Fisher Scientific (Waltham, USA). Reference genes, including *β-actin*, *ef1a*, and *gapdh*, were utilized for normalization (26–28).

### 2.5 Polyclonal antibody preparation for On-NCCRP1

The complete sequence of nonspecific cytotoxic cell receptor protein 1 in Nile tilapia *Oreochromis niloticus* (On-NCCRP1) was previously reported in our 2020 study (30). Subsequently, the ORF sequence of *On-NCCRP1* was amplified using PCR with specific primers that incorporated the *BamH* I and *Xho* I restriction sites (**Supplementary Table S1**). The purified DNA fragments were then ligated into the predigested pGEX-6P-1 plasmid (BT Lab, Wuhan, China) and introduced into BL21 (DE3) chemically competent cells (TransGen, Beijing, China). Positive clones were verified by DNA sequencing and cultured in Luria–Bertani broth supplemented with ampicillin sodium (Amp^+^) at a concentration of 100 µg/mL at 28°C until the OD600 reached 0.6. Isopropyl β-d-thiogalactopyranoside was added to the bacterial culture mixture at a final concentration of 1 μM, and the culture was continued for 8 hours before being harvested by centrifugation. The recombinant protein was then purified using a GST-tag protein purification kit from Beyotime (Shanghai, China), dissolved in sterilized PBS, and confirmed by Coomassie blue staining (**Supplementary Figure S2**).

A rabbit anti-On-NCCRP1 polyclonal antibody was generated using our established protocol beginning in 2023 (31). In summary, two healthy New Zealand White rabbits (~2 kg) were immunized on day 0 with a mixture of On-NCCRP1 protein (400 μg in 750 μL of PBS) and Freund’s complete adjuvant (750 μL) using emulsification. Subsequently, on days 21, 35, and 49, the rabbits were immunized with On-NCCRP1 protein (300 μg in 750 μL of PBS) and Freund’s incomplete adjuvant (750 μL) again. On day 57, the sera of the rabbits were collected, and the antibodies were purified through an affinity chromatography assay using protein A/G agarose beads.

### 2.6 Head kidney leukocyte preparation

HKL preparation was carried out following previous research from 2020 to 2023 (25, 31, 32). In brief, healthy fish were collected and sacrificed. The head kidney was carefully separated, and the neurochords were removed. The head kidney tissue was then cut and passed through a 40-μm cell strainer (Greiner Bio-One GmbH, Frickenhausen, Germany). The resulting cell suspension was placed in Leibovitz’s L-15 medium (Thermo Fisher Scientific, Waltham, USA). The cells were layered onto a 34%/51% Percoll gradient (Solarbio, Beijing, China) and centrifuged using a swing rotor (400 × g, 40 minutes, 4°C). Subsequently, the cells located at the surface of the 51% Percoll layer were gently aspirated, collected via centrifugation, washed, and resuspended in PBS for use in subsequent experiments.

### 2.7 Protein extraction and Western blot

Total protein from HKLs and brain components was extracted with a protein extraction kit (BC3710, Solarbio, Beijing, China). Subsequently, 10 μg of protein sample was loaded onto a 12% SDS−PAGE gel and transferred to a PVDF membrane (Merck, Darmstadt, Germany). The membrane was then blocked with a quick blocking buffer (Beyotime, Shanghai, China) for 10 minutes at room temperature, followed by incubation with a primary antibody, rabbit anti-On-NCCRP1, at a dilution ratio of 1:2000 for 1 hour. After the membranes were washed three times with Tris-buffered saline containing 0.1% Tween-20 (TBST), they were incubated with a secondary antibody, HRP-labeled goat anti-rabbit IgG (H+L) (A0208, Beyotime, Shanghai, China), at a dilution ratio of 1:1000 for 30 minutes. After another round of three washes, the antigen−antibody complexes were detected using the DAB Horseradish Peroxidase Color Development Kit (P0203, Beyotime, Shanghai, China). In addition, a rabbit anti-β-actin monoclonal antibody (dilution ratio of 1:20000) (AC026, ABclonal, Wuhan, China) was used to determine the abundance of the reference protein, β-actin, which was used as a loading control. Moreover, the positive bands obtained from the western blot analysis were transformed into gray values by ImageJ (version 1.54g).

### 2.8 Hematoxylin and eosin staining and immunofluorescence

For the hematoxylin and eosin (H&E) staining and immunofluorescence (IF) assay, the procedures described in our previous work (25, 33) were followed. Briefly, the whole brains of healthy fish were isolated and fixed in Dietrich’s fixative for 24 hours. The brain was then dehydrated in a series of graded alcohol solutions, cleared in xylene, and embedded in paraffin wax. Serial sections (8 μm thick) were rehydrated, stained with an H&E staining kit (C0105S, Beyotime, Shanghai, China), and observed under a microscope.

Selective sections representing typical structures (25) were rehydrated, followed by heat-induced antigen retrieval using a matched solution (P0085, Beyotime, Shanghai, China). The samples were then blocked and incubated with the primary antibody rabbit anti-On-NCCRP1 (dilution ratio of 1:200) for 1.5 hours. After five washes with PBS, the samples were incubated with the secondary antibody Cy3 goat anti-rabbit IgG (H+L) (dilution ratio of 1:500) (AS007, ABclonal, Wuhan, China) for one hour. Finally, the samples were observed and photographed after staining the cell nucleus with 2-(4-amidinophenyl)-6-indolecarbamidine dihydrochloride (DAPI).

### 2.9 Statistical analysis

All the data are presented as the means ± standard deviations (SDs). Tukey’s HSD test was utilized to analyze significant differences through Prism software (version 8.0), and significant differences (*p* < 0.05) are indicated by different letters.

### 2.10 Drawings

TB tools (version 1.108) were used to construct heatmaps. Adobe Photoshop CC (San Jose, CA, USA) and Adobe Illustrator (San Jose, CA, USA) were used to construct and design the final panel.

## 3 Results

### 3.1 Overview of the mRNA expression profile of tilapia brain

**Figure 1:**
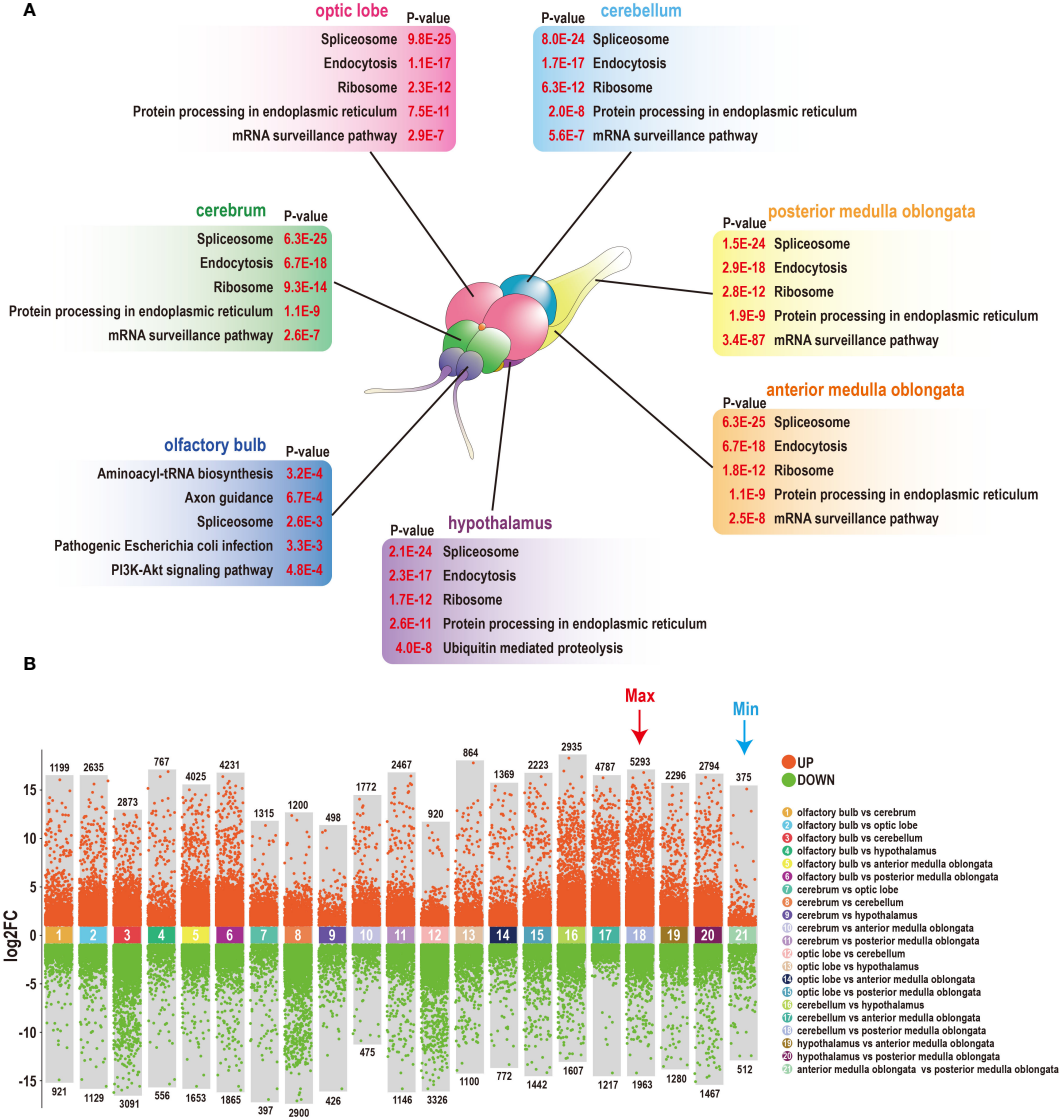
Heterogeneity of seven components of the tilapia brain. **(A)** Top five KEGG annotations of seven components of the tilapia brain. A diagram of the tilapia brain was generated in our previous study (25). **(B)** The number of DEGs among seven components of the tilapia brain was obtained through 21 pairwise comparisons. A total of 75579 DEGs were screened, including the most DEGs that existed between the cerebellum and posterior medulla oblongata, and the fewest DEGs were detected between the anterior and posterior medulla oblongata.

A study was conducted to examine the mRNA expression profiles of seven brain components in tilapia. An RNA-Seq assay was utilized, resulting in the generation of 21 transcriptome libraries. These libraries generated a total of 1240.4 megabases of clean reads, with an average Q30 score of 95.1% and an average mapping ratio of 95.5% to the tilapia genome. Next, a thorough analysis of the KEGG annotations was performed. The top five significantly enriched items in each component were found to be highly similar, with processes mainly related to RNA processing and endocytosis. However, the olfactory bulb was primarily associated with tRNA synthesis and synapse development (**Figure 1A**). A comprehensive analysis of 21 pairwise comparisons resulted in the identification of more than 70000 DEGs. The most substantial differences were observed between the cerebellum and posterior medulla oblongata, with a total of 7256 DEGs. Conversely, the fewest differences were detected between the two segments of the medulla oblongata, with a total of 887 DEGs (**Figure 1B**).

### 3.2 Many immune-related markers are located in the medulla oblongata

**Figure 2:**
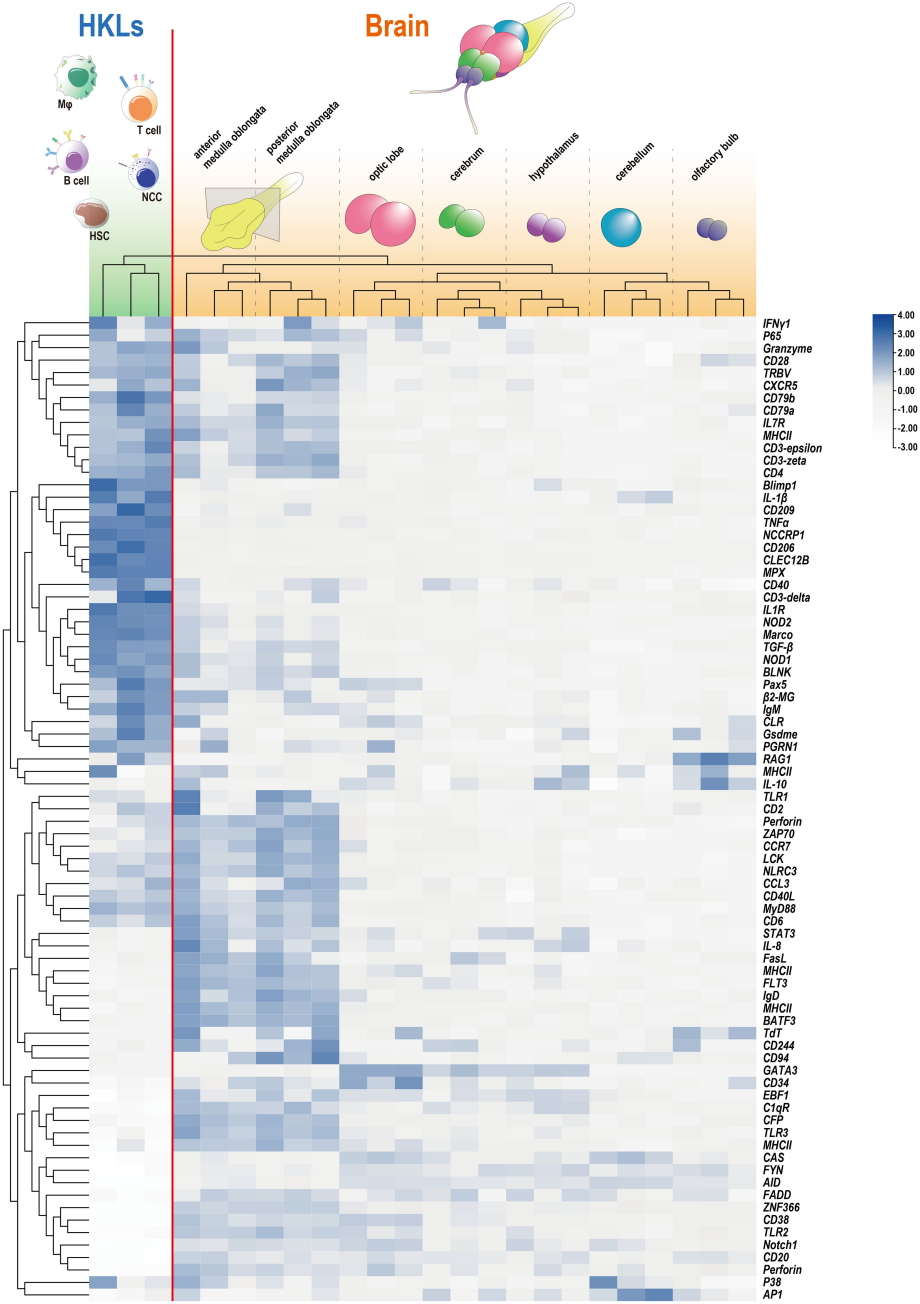
Expression profiles of immune-related genes in HKL and seven components of the tilapia brain detected through RNA-Seq. The RNA-Seq data for tilapia HKL were reported in our previous study (25), and the adjusted FPKM values for HKL and the brain were clustered and presented through a heatmap. The outermost branched sample (HKL) and the remainder (brain) were distinguished by a red line.

To further examine the presence and distribution of lymphocytes marked by specific markers, the abundances of numerous common immune-related markers were gathered and clustered within the brain and tilapia HKL. The analysis revealed that while HKL exhibited a substantial presence of 79 markers, with a mean FPKM value of approximately 64.4, the brain displayed a lower average level of expression, approximately 75% lower, with a mean FPKM value of approximately 16.1 (**Figure 2**). However, in contrast to other brain components, the medulla oblongata exhibited a greater expression level, with a mean FPKM value of approximately 39.6. Furthermore, the medulla oblongata was clearly distinguished and clustered separately from the other brain components. Notably, a total of 27 immune-related markers were more abundant in the medulla oblongata than in the HKL. Among these markers were several typical T-cell markers, such as *CD2*, *CD6*, *ZAP70*, and *LCK* (**Figure 2**).

**Figure 3:**
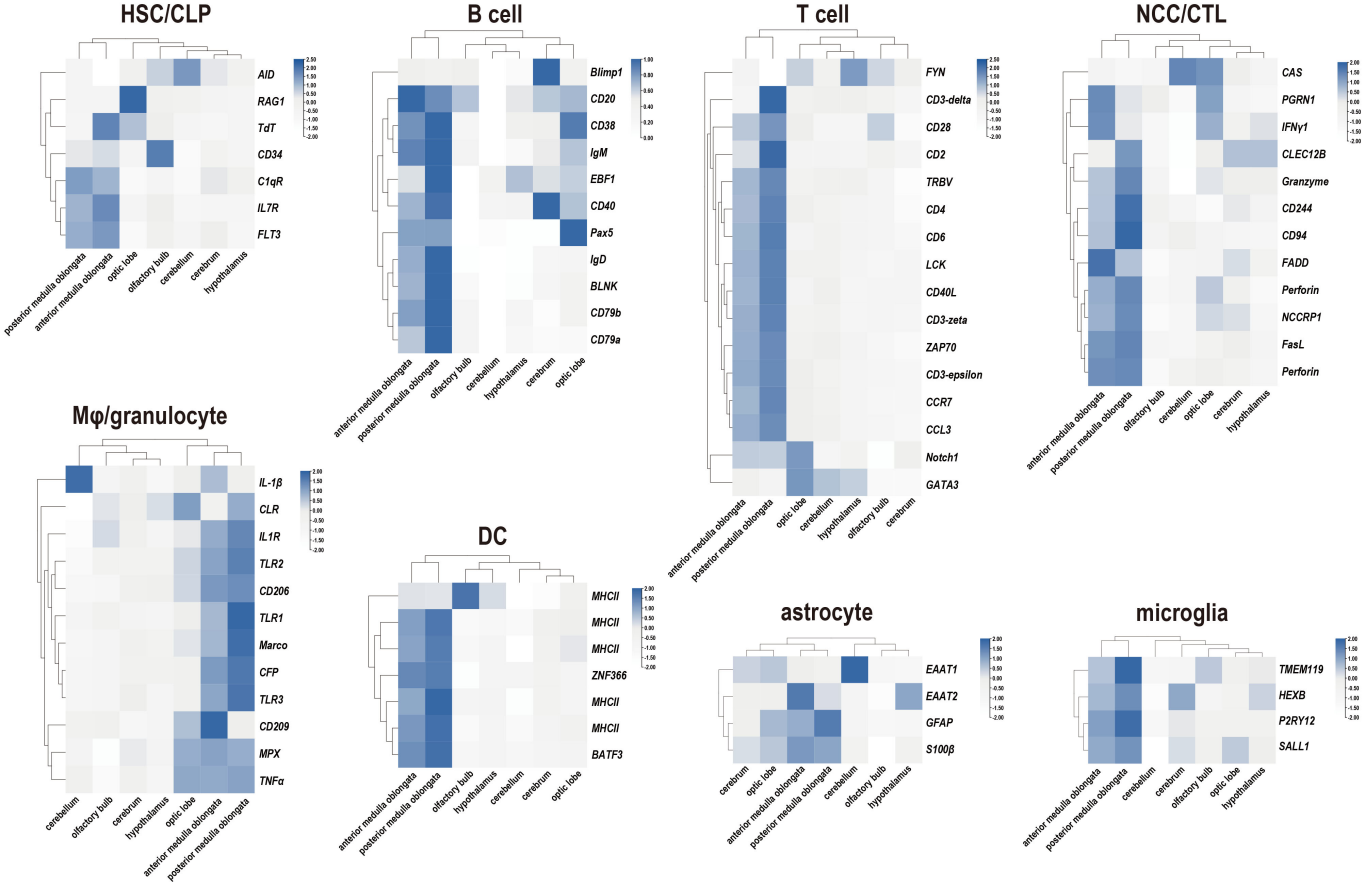
Expression profiles of immune-related markers in seven components of the tilapia brain. Heatmaps showing the clustering of acknowledged marker genes for tilapia lymphocyte subpopulation identification and cerebral defender characteristics, including hematopoietic stem cells (HSCs) or common lymphoid progenitors (CLPs), B cells, T cells, nonspecific cytotoxic cells (NCCs) or cytotoxic T cells (CTLs), macrophages (Mφ) or granulocytes, dendritic cells (DCs), astrocytes, and microglia.

The distribution of markers associated with professional lymphocytes in tilapia (25, 32, 34) was initially analyzed. This analysis included hematopoietic stem cells (HSCs) or common lymphoid progenitors (CLPs), B cells, T cells, nonspecific cytotoxic cells (NCCs) or cytotoxic T cells (CTLs), macrophages (Mφ) or granulocytes, and dendritic cells (DCs). The findings indicated that these molecules were predominantly present in the medulla oblongata, with the exception of markers for macrophages or granulocytes, which are also widely distributed in the optic lobe (**Figure 3**). Additionally, the expression of crucial markers involved in brain surveillance and protection (3, 8, 35, 36), such as *GFAP* for astrocytes and *TEME119* for microglia, was examined, revealing their prominent presence in the medulla oblongata (**Figure 3**).

### 3.3 Inflammation and immune responses in the brain are induced by *S. agalactiae* infection

**Figure 4:**
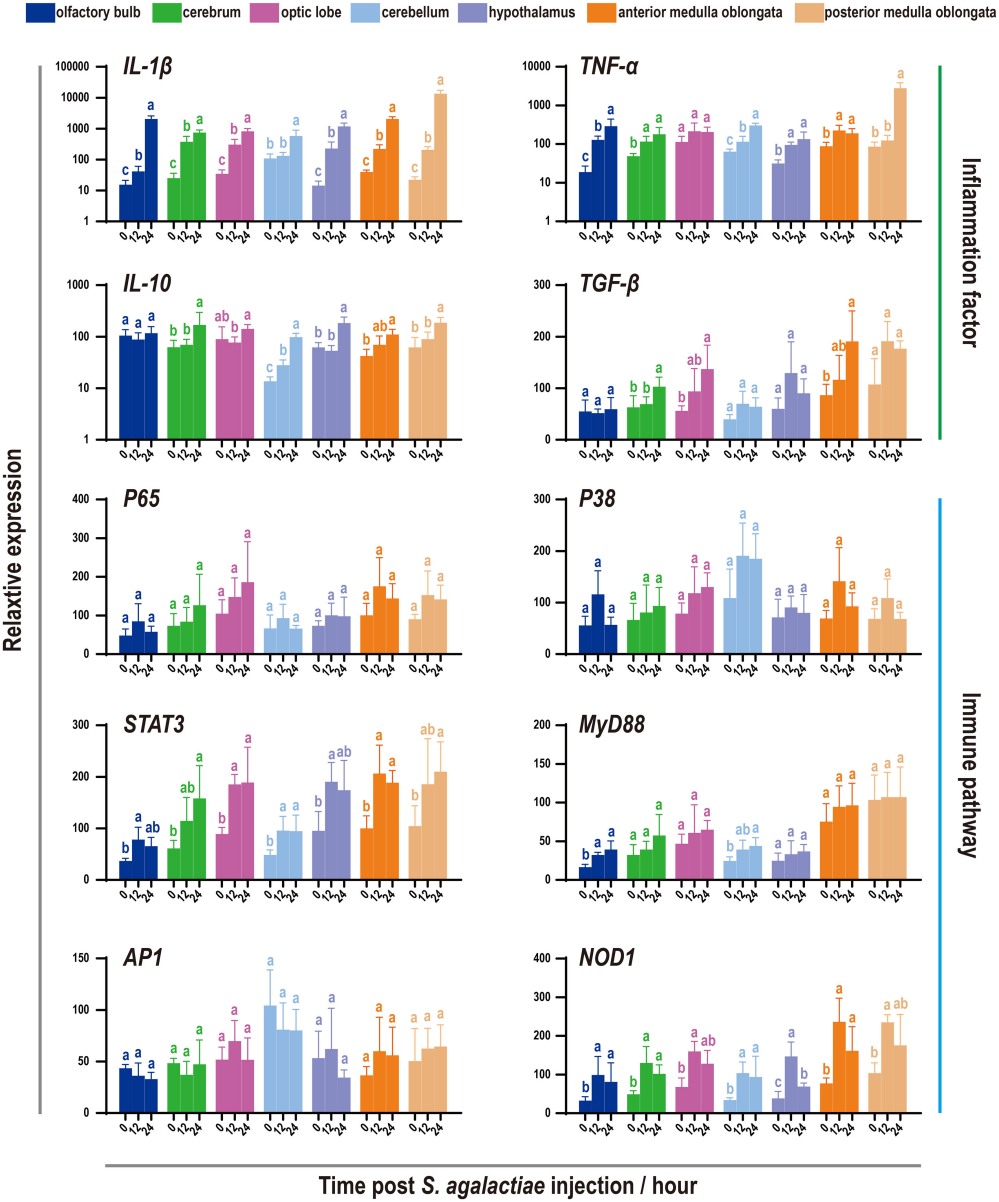
Expression patterns of inflammatory factors and immune pathways associated with seven components of the tilapia brain after *S. agalactiae* infection. The relative expression of four inflammatory factors and six key genes involved in different immune pathways was detected via qRT−PCR. For each given gene, the expression level of the seven components of the tilapia brain at 0 h with the most abundant transcripts was set as 100 to calculate the relative expression of the remaining samples. Different letters indicate significant differences (*p* < 0.05).

To characterize the immune responses of the tilapia brain, a challenge test was performed through *S. agalactiae* injection (**Figure 4**). This bacterium is known to be capable of penetrating the blood−brain barrier and causing severe meningitis (21, 37). Subsequently, the levels of inflammatory factors and key genes involved in various immune pathways were evaluated using qRT−PCR. Notably, robust activation of inflammation was observed, as evidenced by the significant upregulation of the proinflammatory factors *IL-1β* and *TNF-α*. Importantly, the medulla oblongata exhibited the most pronounced increase in proinflammatory factors during bacterial infection, with levels reaching several hundred times the baseline. However, in contrast to those of proinflammatory factors, the responses of the anti-inflammatory factors *IL-10* and *TGF-β* were relatively subdued, limited to certain brain components, and less intensive. Next, the expression patterns of six genes involved in the corresponding immune pathways were evaluated, but only limited activation was detected, primarily in the candidate genes *STAT3*, *MyD88*, and *NOD1* (**Figure 4**).

### 3.4 Nonspecific cytotoxic cell markers in the tilapia brain were strongly activated under *S. agalactiae* infection

**Figure 5:**
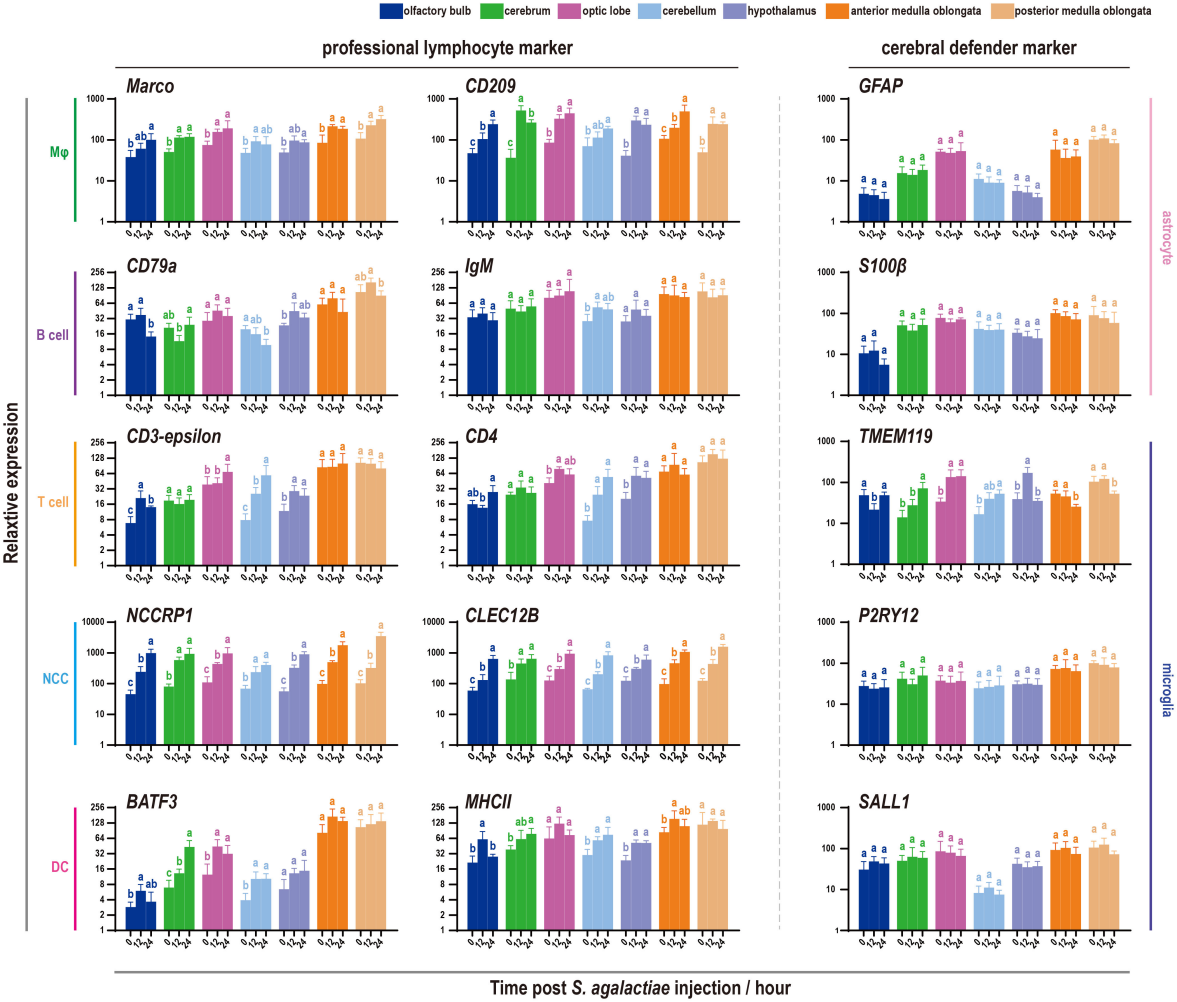
Expression patterns of immune-related markers in seven components of the tilapia brain after *S. agalactiae* infection. The relative expression of ten markers belonging to five types of lymphocytes and astrocyte and microglial markers was detected via qRT−PCR. For each given gene, the expression level of the seven components of the tilapia brain at 0 h with the most abundant transcripts was set as 100 to calculate the relative expression of the remaining samples. Different letters indicate significant differences (*p* < 0.05).

Given the expression patterns of defense markers previously identified in the tilapia brain, a subsequent investigation was conducted to determine the specific types of cells involved in bacterial infection (**Figure 5**). The trends of 10 universal markers belonging to five types of tilapia professional lymphocytes were analyzed, revealing widespread promotion following *S. agalactiae* injection. Notably, *NCCRP1*, the highest marker in the fish NCC subpopulation, displayed a significant and sharp increase under bacterial infection. A similar phenomenon was observed with *CLEC12B*, a potential marker of tilapia NCCs (32, 38). However, the candidates commonly used for adaptive immune cell characterization, such as *CD79a* and *IgM* for B cells and *CD3* and *CD4* for T cells, were minimally influenced by bacterial infection. Additionally, little change in the expression of astrocyte and microglial markers was observed, except for a marker of microglia (*TMEM119*).

### 3.5 NCCs (NCCRP1-positive cells) are widespread in the tilapia brain

**Figure 6:**
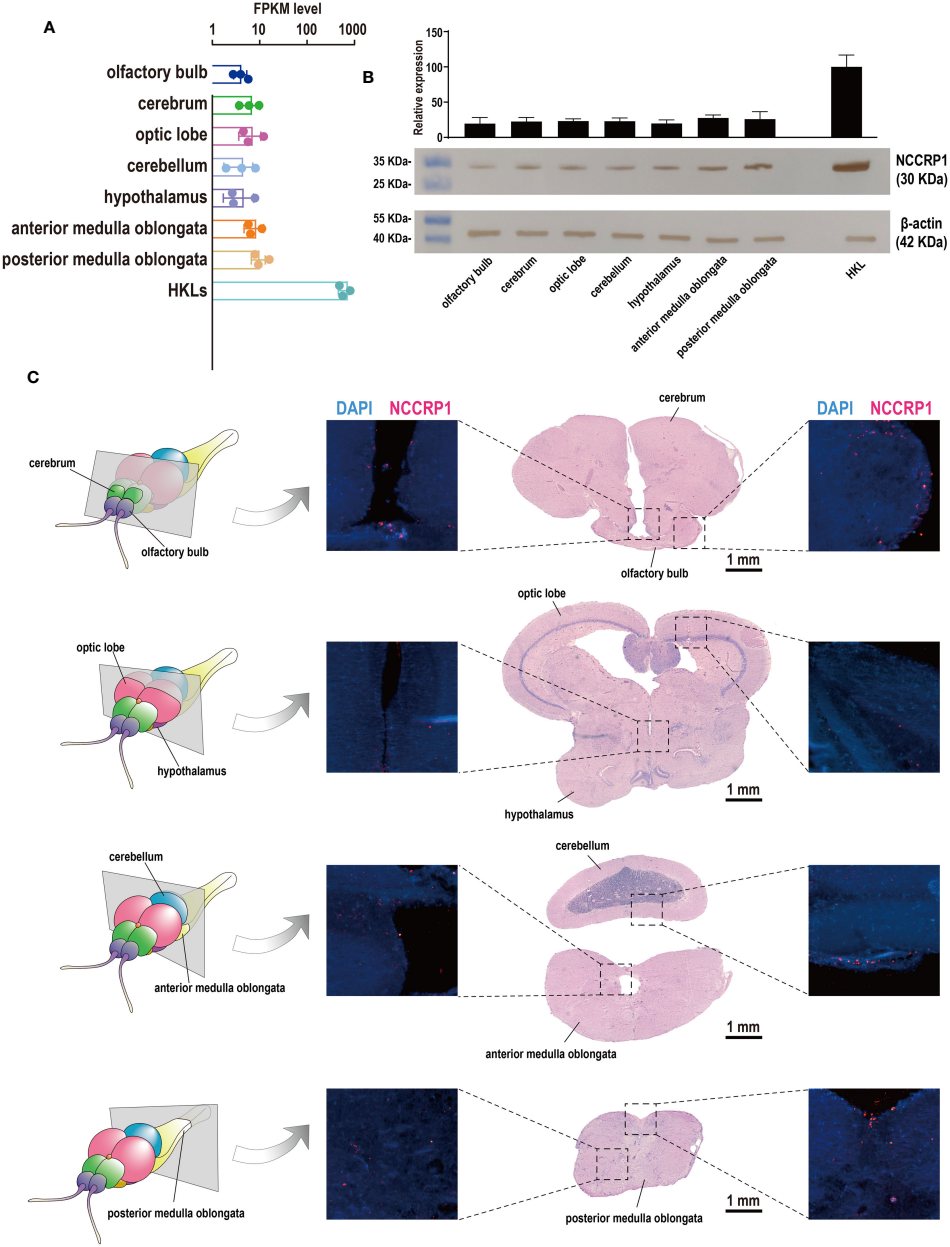
Expression pattern of NCCRP1 in the tilapia brain. **(A)** Relative expression of *NCCRP1* in HKL and seven components of the tilapia brain, as detected through RNA-Seq. **(B)** The statistical analysis of the corresponding gray values was measured by ImageJ software from three parallel tests (**Supplementary Table S2**) and the Western blot analysis of NCCRP1 in HKL and seven components of the tilapia brain; β-actin was used as a reference protein. **(C)** Location of NCCRP1 in the tilapia brain. The left column shows a diagram of sections with typical brain components (25). The corresponding H&E staining results and the presence of NCCRP1 (red) and nuclei (blue) in each section detected by IF are displayed on the right.

Although previous studies demonstrated the presence of *NCCRP1* transcripts in the tilapia brain, with induction evident during bacterial infection (30), the distribution of NCCs (NCCRP1-positive cells) in the tilapia brain remains unclear. Therefore, a prokaryotically expressed recombinant protein, tilapia NCCRP1, was prepared (**Supplementary Figure S2**), and a corresponding rabbit anti-NCCRP1 polyclonal antibody was obtained. The NCCRP1 protein in the tilapia brain and HKLs was assessed using a Western blot analysis, and the results are shown in **Figure 6B**. The details of the *NCCRP1* transcripts in the tilapia brain and HKLs, as evaluated through RNA-Seq, are displayed in histograms (**Figure 6A**). Both the quantification of NCCRP1 at the RNA and protein levels indicated that NCCRP1 was highly abundant in tilapia HKL and widely distributed throughout the brain. Subsequently, discernible positive signals for the NCCRP1 protein were observed in the olfactory bulb, cerebellum, and medulla oblongata (**Figure 6C**). These positive signals were predominantly located on the surface of these tissues, with scarce signals observed in other brain components.

### 3.6 Cytotoxicity in the brain was induced under *S. agalactiae* infection

**Figure 7:**
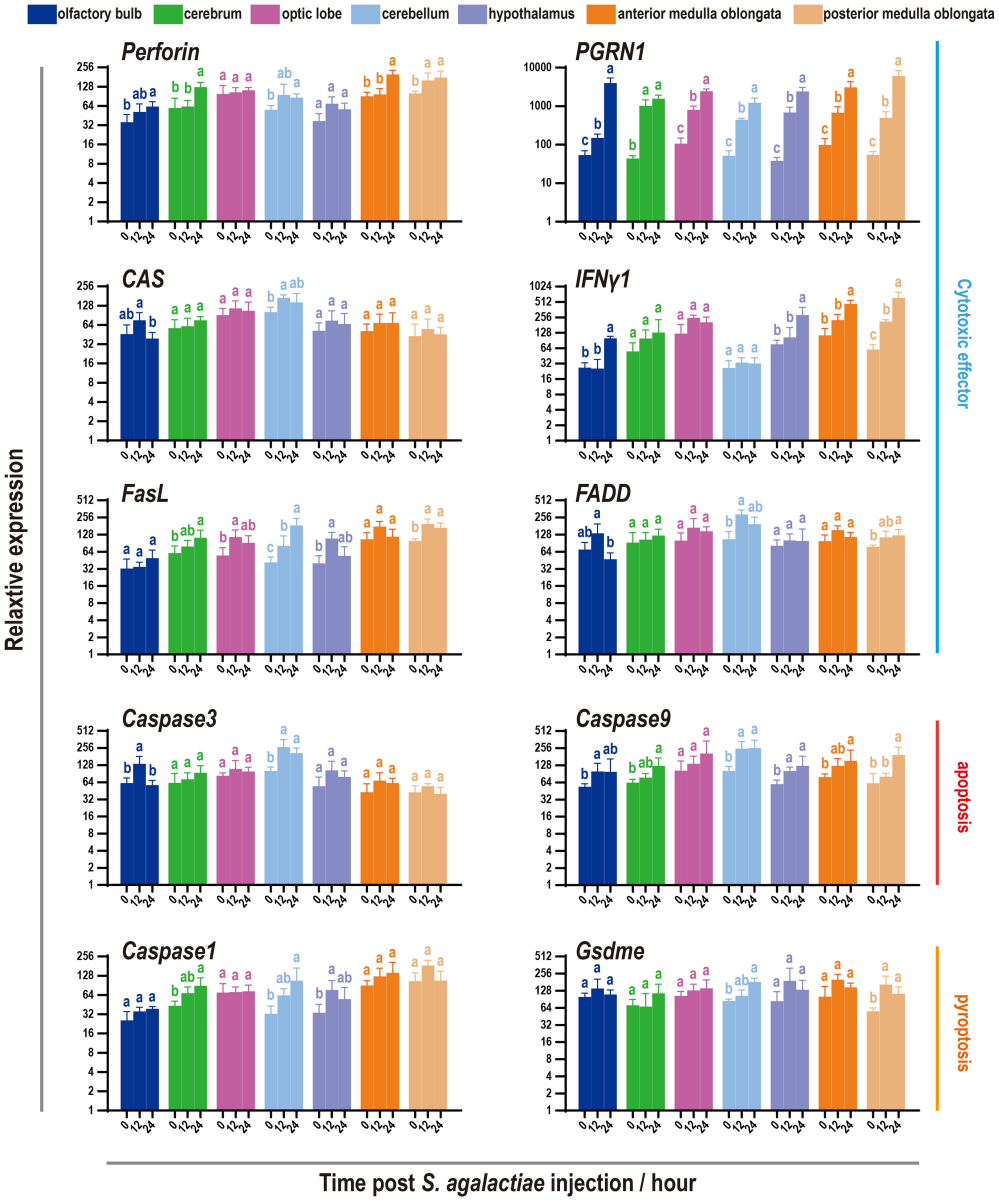
Expression pattern of cytotoxic factors and markers of apoptosis and pyroptosis in seven components of the tilapia brain subjected to *S. agalactiae* infection. The relative expression of six cytotoxic factors and four markers of the apoptosis/pyroptosis process was detected via qRT−PCR. For each given gene, the expression level of the seven components of the tilapia brain at 0 h with the most abundant transcripts was set as 100 to calculate the relative expression of the remaining samples. Different letters indicate significant differences (*p* < 0.05).

Since the discovery of increasing cytotoxic effects mediated by NCCs or NCCRP1 protein in 2021 and 2022 (39, 40), it has become necessary to examine the cytotoxic effects and cell death levels during *S. agalactiae* infection. The findings showed that six common cytotoxicity effectors were activated to varying degrees, with the perforin and interferon genes being particularly prominent (**Figure 7**). However, the activation of apoptosis executors (*Caspase3* and *Caspase9*) and pyroptosis executors (*Caspase1* and *GsdmE*) was minor, while the most significant activation was observed in the cerebellum and medulla oblongata.

## 4 Discussion

Our current study sheds light on the distribution of defenders in the brain and response strategies against *S. agalactiae* infection. To achieve this goal, we first utilized the classical RNA-Seq assay to assess heterogeneity in the tilapia brain. Although there were few differences in the seven brain components identified through KEGG annotation, which could be attributed to the limited resolution of bulk RNA-seq technology and the dominance of neurons in the brain (41–43), the noticeable discrepancy in the number of DEGs emphasized the significant heterogeneity present.

Consequently, a comparison was made between the abundance of professional or peripheral lymphocyte markers present in both the brain and HKL. Astonishingly, the results revealed exceptionally high expression levels of immune-related markers in the medulla oblongata, surpassing even those in the HKL. This is particularly noteworthy considering that the head kidney of teleosts has traditionally been regarded as the primary hematopoietic tissue and central immune organ (16, 17), and tilapia HKLs were found to be almost exclusively composed of professional immune cells (25, 32, 34). The presence of such a large number of professional lymphocytes in the medulla oblongata strongly suggests frequent migration under steady-state conditions, similar to the situation in mammals (3). However, there are some differences to note, as the main immunological niches in the mammalian brain are located in the CP, meninges, and CSF (3). Although CP has been reported in the zebrafish myelencephalon, the obvious positive signal was limited to the saccus dorsalis zone of adult brains rather than the medulla oblongata (44). In contrast, bony fish have been found to have a high flow of CSF around the medulla oblongata (45), which likely performs the principal transport function and may represent the primordial form. Additionally, in the tilapia medulla oblongata, the majority of cerebral defender microglia and astrocytes were detected. However, conflicting results have been recorded in zebrafish, as more microglia were found in the optic lobe (46, 47). Nevertheless, similar to tilapia, dense astrocytes have also been observed in the medulla oblongata of zebrafish, and these astrocytes are associated with synapses and interact with each other (48). These findings collectively underscore the immunological significance of the medulla oblongata in the tilapia brain.

Furthermore, challenge tests were conducted and evaluated, revealing evident inflammation in the brain occurring no later than 12 hours after injection, which aligns with previous findings (27, 33, 49). Moreover, relatively moderate activation of the immune pathway was also observed, similar to prior reports that the intense response of these candidates mainly occurs in the head kidney and spleen of tilapia (49). This may be associated with the immune tolerance of the brain, which serves to prevent excessive cell death (2, 50).

Notably, the most significant response in the tilapia brain under *S. agalactiae* infection was observed in the NCCs, followed by Mφ, as indicated by the universal marker genes. In particular, the expression levels of NCC markers during infection were significantly greater than those under normal physiological conditions. NCCs, which are considered potential evolutionary precursors of natural killer (NK) cells, possess spontaneous recognition and binding capabilities against pathogenic molecules and xenogeneic tissues, resulting in the elimination of specific target cells (30). However, the morphology and molecular markers of NCCs in fish differ from those of mammalian NK cells. For instance, the CD56 molecule, which is a core marker of human NK cells (51), is absent in almost all fish species. NCCRP1 was identified as the first recognized marker of NCCs (52). Furthermore, our previous studies reported several potential markers for tilapia NCCs, including *CLEC12B*, which showed complete overlap with *NCCRP1* (25, 32). Similarly, the distribution and response patterns of *CLEC12B* were found to be similar to those of *NCCRP1* (38). In this study, highly consistent profiles of *CLEC12B* were also observed, highlighting the involvement of NCCs in the defense of the tilapia brain against bacterial infections. Additionally, in mammals, proinflammatory factors are secreted by macrophages, dendritic cells, and CD4^+^ T cells (53, 54), and similar colocalization phenomena have been reported in tilapia (25, 34), suggesting the activation of inflammation due to the stimulation of macrophages in the brain.

Therefore, an antibody against tilapia NCCRP1 was prepared, and the NCCs (NCCRP1^+^ cells) in tilapia were examined. These results indicated that NCCRP1 is widely distributed in the tilapia brain, although its abundance remains lower than that in the HKL. However, RNA-Seq data revealed that the rank of *NCCRP1* transcripts more than 60% of all genes in the tilapia brain. The visually poor content may be attributed to the extreme abundance of *NCCRP1* in HKLs, where it exhibited a positive rate of greater than 25% (25). Furthermore, the mean FPKM of *NCCRP1* in HKLs (approximately 3000) was comparable to that of common reference genes such as *GAPDH* and *EF1a*. Subsequently, abundant NCCRP1^+^ cells were detected in the tilapia brain, particularly on the surfaces of the olfactory bulb, cerebellum, and medulla oblongata, suggesting that the meninges in fish might have similar immunological functions to those in mammalian brains, acting as neuroimmune interface (3, 13). Finally, cytotoxicity and cell death were assessed and confirmed, and the results indicated that these effects were likely induced by NCCs (39, 40), further supporting the involvement of NCCs in dominant antibacterial immunity in the tilapia brain.

In summary, our study provides insight into the distribution of professional and autochthonous defenders in the tilapia brain and identifies the primary immune cells involved in combating streptococcal infection. These findings suggest that the complex neuroimmune connection observed in mammals was already emerging in the tilapia brain, with NCCs playing a central role. This research significantly contributes to our understanding of the evolutionary development of neuroimmune mechanisms from fish to humans.

## Data availability statement

The datasets presented in this study can be found in online repositories. The names of the repository/repositories and accession number(s) can be found below: https://www.ncbi.nlm.nih.gov/, PRJNA1084852.

## Ethics statement

The animal study was approved by Ethics Committee of Guangdong Ocean University. The study was conducted in accordance with the local legislation and institutional requirements.

## Author contributions

XH: Conceptualization, Funding acquisition, Writing – original draft, Writing – review & editing. QL: Conceptualization, Data curation, Investigation, Methodology, Writing – original draft, Writing – review & editing.
