# Supplementary figures and images for: Medulla oblongata and NCCs are central defenders against Streptococcus agalactiae infection of the tilapia brain
Source: Front Immunol. 2024 Jul 1;15:1442906. doi: 10.3389/fimmu.2024.1442906 (PMC11246860; doi:10.3389/fimmu.2024.1442906)

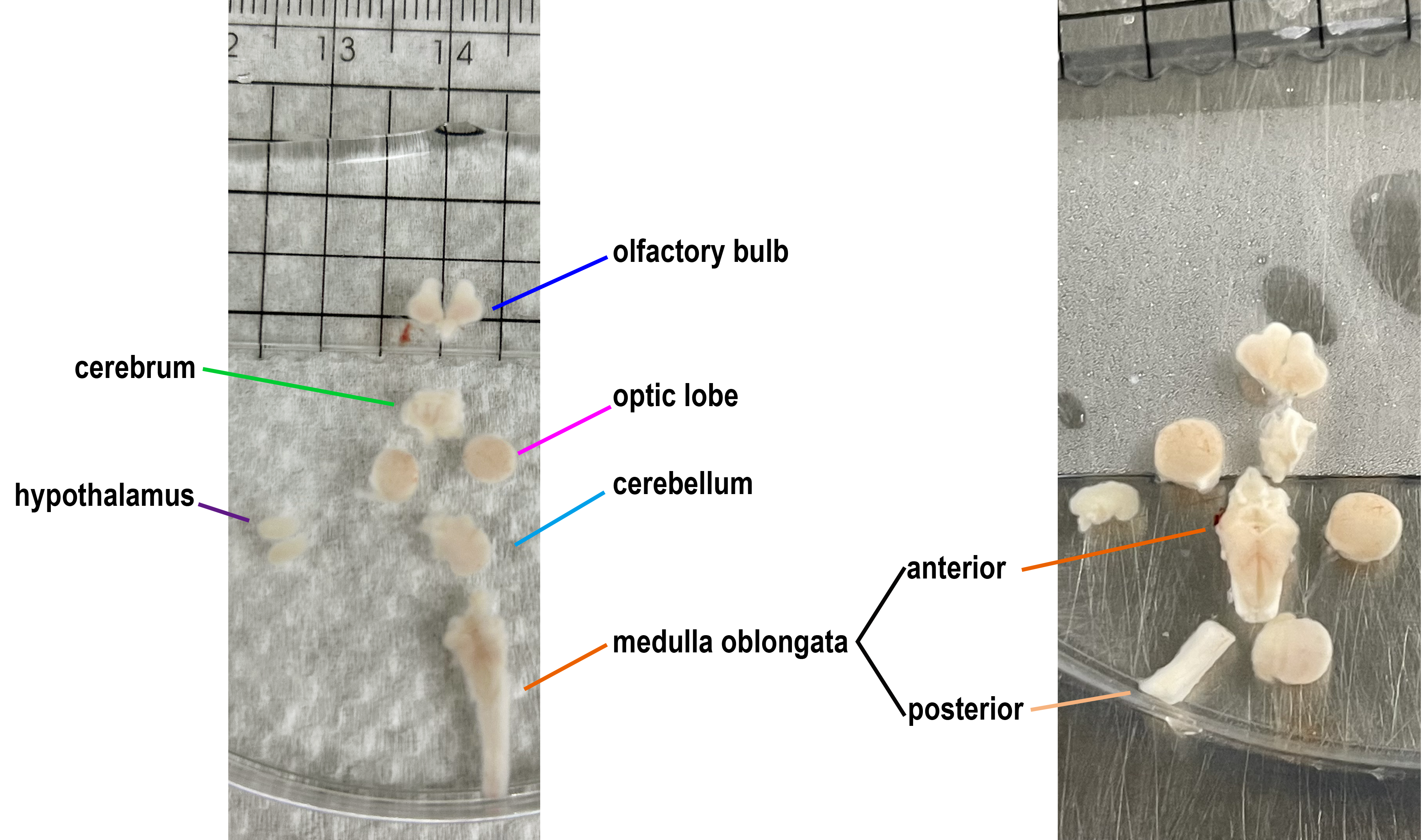

Supplement: Supplementary file 1 [file DataSheet_1.zip › supplementary materials/Fig.S1. Seven components of tilapia brain.tif]

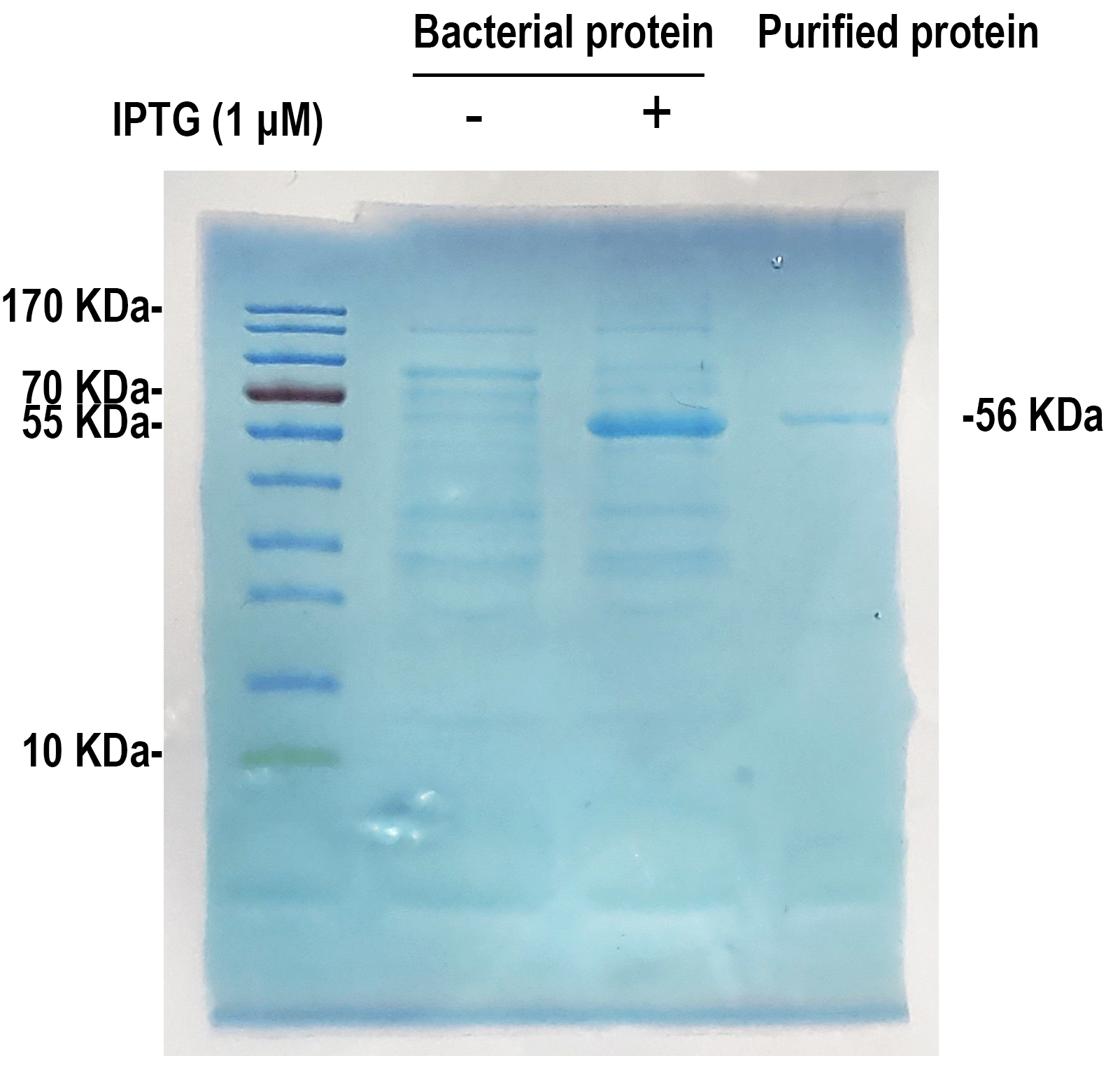

Supplement: Supplementary file 1 [file DataSheet_1.zip › supplementary materials/Fig.S2. Coomassie blue stain of recombinant protein.tif]
